# Effects of synaptic and intrinsic parameters in shaping dynamic responses to olfactory input: a combined computational-experimental study of two glomerular microcircuits

**DOI:** 10.1186/1471-2202-12-S1-P365

**Published:** 2011-07-18

**Authors:** William Erik Sherwood, Ryan Carey, Matt Wachowiak

**Affiliations:** 1 Center for BioDynamics, Boston University, Boston, MA 02215, USA; 2 Department of Biomedical Engineering, Boston University, Boston, MA 02215, USA; 3 Department of Physiology and Brain Institute, University of Utah, Salt Lake City, UT 04108, USA

## 

Initial synaptic processing of odors occurs in the mammalian olfactory bulb (OB): temporally dynamic odorant-evoked inputs are first processed at the olfactory receptor neuron (ORN) level, and similarly dynamic, patterned output is transmitted from the mitral cell (MC) layer to olfactory cortex. Prior work has shown that bursts of odor-evoked ORN activity exhibit odor-specific and glomerulus-specific durations, rise times, latencies, and amplitudes [1]. Similarly diverse patterning is seen in MC activity, which is temporally organized around the respiratory cycle and changes qualitatively during odor sampling. The temporal spread of sensory input following a single inhalation (~100-300 ms) is comparable to the range of discrimination times for different olfactory tasks [2,3], consistent with these dynamics being important in shaping odor perception.

Situated between the ORN input and MC output layers, the neuronal circuitry of the glomerular layer acts to consolidate and modulate OB output. Thousands of juxtaglomerular neurons from three distinct classes form the neuronal network within a glomerulus; several synaptic and gap junctional microcircuits have been identified in the intraglomerular network [4]. What transformation(s) of ORN input into MC output might the intraglomerular circuitry perform? We investigate temporal dynamics in computational models of two intraglomerular microcircuits: the classical ORN-MC circuit and a variant circuit that incorporates between the ORN and MC an external tufted (ET) cell capable of endogenous bursting [5]. These neurons are represented with single-compartment, Hodgkin-Huxley-style models [6,7]. Circuit inputs are calcium signals recorded from the presynaptic terminals of ORNs of head-fixed rats exposed to odorants using a ‘sniff playback’ mechanism [8,9]. These calcium signals are converted to excitatory synaptic inputs with temporal signatures closely matching that of inputs to the real neurons. The response dynamics of the circuits’ MC output are strongly shaped by the input signal. We explore how the circuits’ dynamics vary for different odorants, synaptic strengths, and degrees of synaptic adaptation, and we compare the two circuits’ dynamics as parameters controlling intrinsic properties of the ET and MC cells are varied.
